# *Desmophyllum dianthus* (Esper, 1794) in the Scleractinian Phylogeny and Its Intraspecific Diversity

**DOI:** 10.1371/journal.pone.0050215

**Published:** 2012-11-28

**Authors:** Anna M. Addamo, James D. Reimer, Marco Taviani, André Freiwald, Annie Machordom

**Affiliations:** 1 Departmento de Biodiversidad y Biología Evolutiva, Museo Nacional de Ciencias Naturales Consejo Superior de Investigaciones Científicas, Madrid, Spain; 2 Rising Star Program Trans-disciplinary Organization for Subtropical Island Studies, University of the Ryukyus, Nishihara, Okinawa, Japan; 3 Istituto di Scienze Marine, Consiglio Nazionale delle Ricerche, Bologna, Italy; 4 Biology Department, Woods Hole Oceanographic Institution, Woods Hole, Massachusetts, United States of America; 5 Department for Marine Research, Senckenberg am Meer, Wilhelmshaven, Germany; King Abdullah University of Science and Technology, Saudi Arabia

## Abstract

The cosmopolitan solitary deep-water scleractinian coral *Desmophyllum dianthu*s (Esper, 1794) was selected as a representative model species of the polyphyletic Caryophylliidae family to (1) examine phylogenetic relationships with respect to the principal Scleractinia taxa, (2) check population structure, (3) test the widespread connectivity hypothesis and (4) assess the utility of different nuclear and mitochondrial markers currently in use. To carry out these goals, DNA sequence data from nuclear (ITS and 28S) and mitochondrial (16S and COI) markers were analyzed for several coral species and for Mediterranean populations of *D. dianthus*. Three phylogenetic methodologies (ML, MP and BI), based on data from the four molecular markers, all supported *D. dianthus* as clearly belonging to the “robust” clade, in which the species *Lophelia pertusa* and *D. dianthus* not only grouped together, but also shared haplotypes for some DNA markers. Molecular results also showed shared haplotypes among *D. dianthus* populations distributed in regions separated by several thousands of kilometers and by clear geographic barriers. These results could reflect limited molecular and morphological taxonomic resolution rather than real widespread connectivity. Additional studies are needed in order to find molecular markers and morphological features able to disentangle the complex phylogenetic relationship in the Order Scleractinia and to differentiate isolated populations, thus avoiding the homoplasy found in some morphological characters that are still considered in the literature.

## Introduction

Deep-sea ecosystems represent the largest biome of the global biosphere [1], [2] in which cold (or deep) water corals (CWC) play a significant ecological role [3]. In spite of this, many fundamental traits of cold-water coral biology still need to be more properly understood. One such deficiency is in the scantiness of studies focusing on the molecular biology of CWCs, which would shed light on their proper supraspecific taxonomic positioning, phylogeography and connectivity.

When morphological features are insufficient to disentangle the evolutionary history of certain taxa, molecular phylogenies (inter- and intraspecific) can provide evidence of past evolutionary events, and allow comparisons of intra- and interpopulation variability to identify patterns of biological diversity (e.g. [4]–[6]). Recently, multidisciplinary approaches have played a strong role in scleractinian systematics [7] (e.g. [8]), but the results of these efforts are not uniform, especially for solitary azooxanthellate CWCs, thereby causing a biased view of the evolutionary history and global biogeography of Scleractinia (e.g. [8]–[12]).

Additional molecular data for more solitary CWC could corroborate the concept that some CWC species are widely distributed. However, recent studies have shown that some of these widespread eurybathic species actually represent multiple genetically distinct cryptic species that can be subdivided by geography or depth [13]–[15].

CWC are widespread in the Mediterranean Sea, occurring as either extant species or as Pleistocene fossils [16]–[18]. For many years, CWC were considered to be near extinction in the Mediterranean Sea until the unexpected rediscovery of living banks of *Lophelia pertusa* and *Madrepora oculata* in Santa Maria di Leuca, in the Ionian Sea [19]. More discoveries of these species followed, with new sites found in the southwestern Adriatic Sea, Strait of Sicily, Catalan-Provencal margin and Alboran Sea [18], [20]–[23].

The current interest in Mediterranean CWC ecosystems necessitates an assessment of the biological status of its major coral components, among which the solitary scleractinian *Desmophyllum dianthus* occupies an important position.

*Desmophyllum dianthus* (Esper, 1794) (syn. *Desmophyllum cristagalli*, according to Cairns & Zibrowius [24]) is a species still considered as cosmopolitan, and specimens have been reported in all oceans of the world from coastal Antarctic to the Arctic Circle. It is a solitary coral, classified in the family Caryophylliidae based on morphological characters. *Desmophyllum dianthus* is a slow-growing coral (0.5–2 mm per year) with a long lifespan (up to 200 years) [25], [26].

*Desmophyllum dianthus* occurs in the upper bathyal zone (common depth range between 200–2500 m; see [16], [27]), associated with frame building species (e.g. *Lophelia pertusa* and *Madrepora oculata*). However, records at shallower depths exist for New Zealand fjords (from 4 m [28]), and Chilean fjords (from 8 m [29]), where *D. dianthus* was found in an unusual symbiosis with the microendolithic phototrophic alga *Ostrobium quecketii* [30].

*Desmophyllum dianthus* also contributes to the reef framework as aggregated colonies or “clumps of specimens” [31]. This species is a preferred target to study oceanographic-climatic variability by deciphering geochemical signals embedded within its skeletal aragonite (e.g. [25], [26], [32]–[34]). Recently, preliminary analyses of the phylogenetics, ecology, including the unusual symbiosis with algae, and reproduction (e.g. [12], [29], [30], [35], [36]) have been conducted for *D. dianthus*, but each topic still needs further investigation to gain comprehensive knowledge about this species. Here, we aim to (1) characterize Mediterranean *D. dianthus* with molecular markers and investigate its phylogenetic relationships with respect to principal scleractinian taxa, (2) undertake the study of the genetic structure of extant populations, (3) validate the widespread connectivity hypothesis and (4) corroborate the utility of nuclear (ITS1-5.8S rRNA-ITS2 and 28S rRNA) and mitochondrial (16S rRNA and COI) markers.

## Methods

### Samples collection, species and study area

Live *D. dianthus* specimens were collected from the Mediterranean Sea at depths between 276–1102 m from living CWC grounds in the Adriatic Sea, Ionian Sea and Strait of Sicily (Fig. S1, Table S1). Specimens analyzed in this study were initially preserved in 80% ethanol at 4°C prior to being stored in absolute ethanol.

This study was based on specimens collected in 2006/2007 and 2009, during scientific cruises M70, SETE06, APLABES, CORSARO, CORAL2002, MARCOS and MEDCOR on board the RV *Meteor* and RV *Urania*. All necessary permits were obtained for the described field studies. The study areas were not marine protected or privately owned areas, and sampling did not require any specific permission. This study did not involve endangered or protected species listed in the IUCN Red List of Threatened Species.

### DNA extraction, PCR amplification and sequencing

Small pieces of tissue were taken from each sample and rinsed with ultrapure water prior to extraction. DNA extraction was performed using the QIAGEN BioSprint 15 DNA Blood Kit (Qiagen Iberia S.L., Madrid) following the manufacturer's instructions, but with an extended period of proteinase K lysis (overnight incubation at 55°C).

For each specimen, the concentration of extracted genomic DNA was measured using a Nanodrop 1000 (Thermo Scientific). Each aliquot was then diluted at a ratio of 1∶20 in 200 µl final volume.

Four partial DNA genes and regions, including nuclear and mitochondrial markers with different rates of mutation, were partially amplified and sequenced: 1) the internal transcribed spacer regions (internal transcribed spacer 1–5.8S ribosomal DNA - internal transcribed spacer 2, hereafter designated ITS), 2) the large ribosomal subunit (28S), 3) the mitochondrial large ribosomal subunit (16S) and 4) the mitochondrial cytochrome oxidase c subunit I (COI).

#### Nuclear genes

Polymerase chain reactions (PCR) were carried out in a total volume of 50 µl, with 1× PCR Buffer (final concentration MgCl_2_ 2 mM), up to 3 mM MgCl_2_ (only for 28S), 0.05 mM of each dNTP, 0.14 µM of each primer, 1.5 U of Taq polymerase and 2 µl of template DNA.

Nuclear ITS was amplified using the primers ITS2.1 and ITS2.2 [37], and a portion of the 5′ end of the nuclear 28S (including the C1 and D2 domains) was amplified using the primers C1′ and D2MAD [38]. PCR amplification was performed on extracted DNA under the following conditions: an initial denaturing step of 4 min at 94°C, followed by 40 cycles of 45 s (ITS) or 1 min (28S) at 94°C, 1 min annealing at 57°C (ITS) or 56°C (28S), and 1 min extension at 72°C and a final extension step of 10 min at 72°C.

The products were visualized under blue light in a 1.5% agarose gel stained with SYBR Safe, and then purified using an ethanol/sodium acetate precipitation method. Both strands were sequenced using BigDye Terminator and an ABI PRISM 3730 DNA Sequencer (Applied Biosystems).

#### Mitochondrial genes

PCR reactions were carried out in 20 µl using the HotStartTaq *Plus* Master Mix Kit (QIAGEN), following the manufacturer's instructions, and with 1 µl of each primer (10 µM each) and 1 µl of template DNA.

16S was amplified using the hard coral specific primers LP16SF and LP16SR [39].

COI was amplified using a novel forward primer COIcoralF (5′-GATCATCTTTATAATTGT-3′) and the reverse primer HCO2 [40]. The COIcoralF primer was specifically designed for Scleractinia with Corallimorpharia sequences as outgroup. The following thermal cycle conditions were utilized: an initial activation step of 5 min at 95°C, followed by 30 cycles of 30 s at 94°C, 30 s at 50°C, and 45 s at 72°C with a final extension step of 10 min at 72°C.

Amplified products were cleaned up by *Exo1/SAP* treatment, with the following thermal cycling conditions: 20 min at 37°C, 30 min at 83°C, and a final hold at 4°C. Both strands were sequenced using BigDye Terminator and an ABI3730XL DNA Sequencer (Applied Biosystems).

For both nuclear and mitochondrial markers, when amplification failed, different dilutions of template DNA up to 1∶500 were used to repeat the PCR.

### Alignments

Sequences were verified and primers were cut from the alignment using the program SEQUENCHER v4.10.1 (Gene Codes Corporation). In order to expand our result to a wide spectrum of families in Scleractinia with special emphasis on Caryophylliidae, we also sequenced specimens for potentially closely related species. Additional previously published nuclear and mitochondrial sequences were also retrieved from GenBank and added to the alignments (Table S2).

The nucleotide sequences of ITS, 28S, 16S and COI were separately aligned in ClustalX [41] using the default settings. The resulting alignments were inspected by eye and manually checked and adjusted with Se-Al v2.0a11 [42]. Consequently, four matrices with the final alignments were generated (available upon request from the corresponding author).

### Phylogenetic analyses

The model of best fits for nucleotide evolution for each final alignment was determined by the Akaike Information Criterion (AIC) in jModelTest [43]. Phylogenetic analyses were performed using PhyML v3.0 [44] for Maximum Likelihood (ML), MrBayes v3.1.2 [45] for Bayesian Inference (BI), and PAUP*v4.0b10 [46] for Maximum Parsimony (MP). The different data sets were analyzed separately and then tested for heterogeneity with PAUP*v4.0b10 between data partitions, before combining the data in a unique matrix. The ML and MP analyses were performed with 1000 bootstrap replicates. For the BI analyses, five double parallel runs were performed for 5 million generations with one cold and three heated Markov Chains Monte Carlo (MCMC) for each run, sampling trees at 1000 generations intervals (5000 trees were saved during MCMC for each run) when the average standard deviation of split frequencies betweens runs was less than 0.01. The addition of more generations (up to 10 million) was necessary for some matrices to reach a standard deviation of split frequencies below 0.01 and for the effective sample size (ESS) to reach the suggested minimum value (>200), as verified using Tracer v1.5 [47]. Ten percent of all trees were discarded as burn-in, and the remaining trees were used to calculate the posterior probabilities. Maximum clade credibility trees were generated by TreeAnnotator [48].

To place *D. dianthus* within one of the Caryophylliidae clades as defined by previous studies [11], [38], [49], [50], nuclear (ITS, 28S) and mitochondrial (16S, COI) sequences were obtained from this study. Additional sequences were downloaded from GenBank (Table S2) for the following caryophylliid genera: *Caryophyllia, Crispatotrochus, Deltocyathus*, *Lophelia*, *Polycyathus*, *Paracyathus*, *Cladocora*, *Rhizosmilia*, *Phyllangia*, *Ceratotrochus*, *Odontocyathus, Vaughanella*, *Thalamophyllia*, *Tethocyathus*, *Solenosmilia*, *Pourtalosmilia*, *Stephanocyathus*, *Trochocyathus*, *Conotrochus*, *Dactylotrochus* and *Dasmosmilia*. Scleractinia genera were recognized based on classification assigned by Cairns and listed in [27]. Taxonomic discrepancies of some genera (e.g. *Cladocora*) are not included in this study.

Representative species of the following families were also included in the analyses: Flabellidae, Dendrophylliidae, Poritiidae, Siderastreidae, Acroporidae and Agariciidae (“complex” corals); Meruliniidae, Pectiniidae, Faviidae, Mussiidae, Pocilloporiidae and Fungiidae (“robust” corals); Meandriniidae, Oculinidae, Astrocoeniidae and Euphylliidae (families with species that can be included in both “complex” and “robust” groups); Micrabaciidae and Gardineriidae (‘basal’ corals as defined by Kitahara *et al.* [11] and Stolarski *et al.* [12]). *Ricordea florida* (Anthozoa: Corallimorpharia) was selected as the outgroup species for all analyses.

### Haplotype Network

Intraspecific phylogenies were evaluated using a network approach. Analyses were performed on 54 *D. dianthus* specimens analyzed for ITS haplotypes (551 bp) and 49 for 16S haplotypes (281 bp) from two geographic regions, the South Pacific Ocean and the Mediterranean Sea. A median-joining network was performed with the software Network 4.610 (Fluxus Technology Ltd), based on ITS and 16S alignments, as they are the only *D. dianthus* sequences well represented in GenBank. The framework for testing evolutionary hypotheses was obtained using neutrality test, genetic polymorphism and gene flow analyses, performed with DnaSP v.5.0 software [51] and Arlequin v3.5.1.2 [52].

## Results

Four alignments were obtained: 1) ITS (112 taxa), 2) 28S (98 taxa), 3) 16S (57 taxa) and 4) COI (51 taxa) (Table 1).

**Table 1:** Lengths of the PCR products and the respective alignments of *Desmophyllum dianthus* and the main scleractinian family taxa (for more details see Fig. 1 and Table S2), along with the best-fit models selected by AIC in Modeltest 3.7.

	ITS	28S	16S	COI	Combined
*PCR product length*	610	759	280	538	-
*Alignment length*	843	791	575	512	2595
*Variable characters*	551 (*653)	208 (*598)	371 (*497)	229	1133
*Parsimony informative characters*	319 (*488)	119 (*293)	235(*390)	204	682
*Model of best fit*	TIMef+G	GTR+I+G	TIM+G	GTR+I+G	GTR+I+G

* Considering gaps as a fifth state of characters.

Taking nuclear (ITS+28S) and mitochondrial (16S+COI) data together, a total of 2595 sites from 44 specimens were analyzed for the four DNA regions utilized in this study.

New sequences obtained in the present study were deposited in GenBank (Table S2).

### Phylogenetic analyses

**Figure 1:**
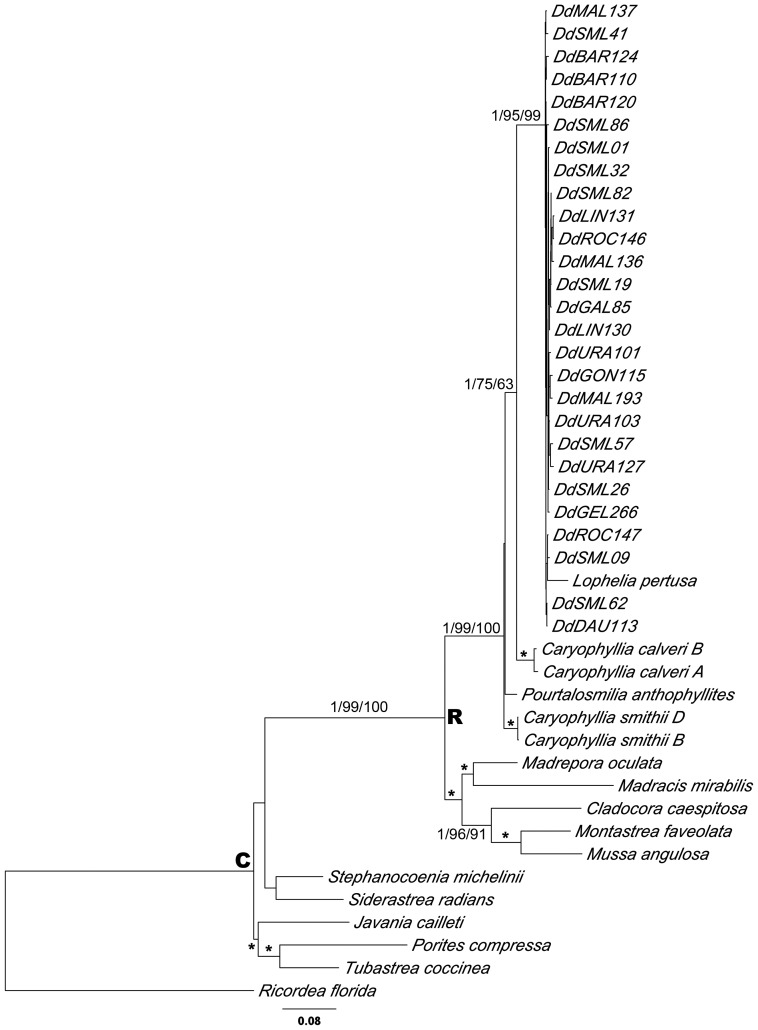
Phylogenetic relationship between *Desmophyllum dianthus* and principal taxa from Scleractinia families. Tree topology was inferred by Bayesian analysis, based on combined mitochondrial and nuclear genes. R and C indicate “robust” and “complex” groups, respectively. Numbers on main branches show the Bayesian posterior probability and bootstrap support obtained under Maximum Parsimony and Maximum Likelihood criteria, respectively. Stars indicate other well-supported clades (pp≥95; bootstrap >70).

**Figure 2:**
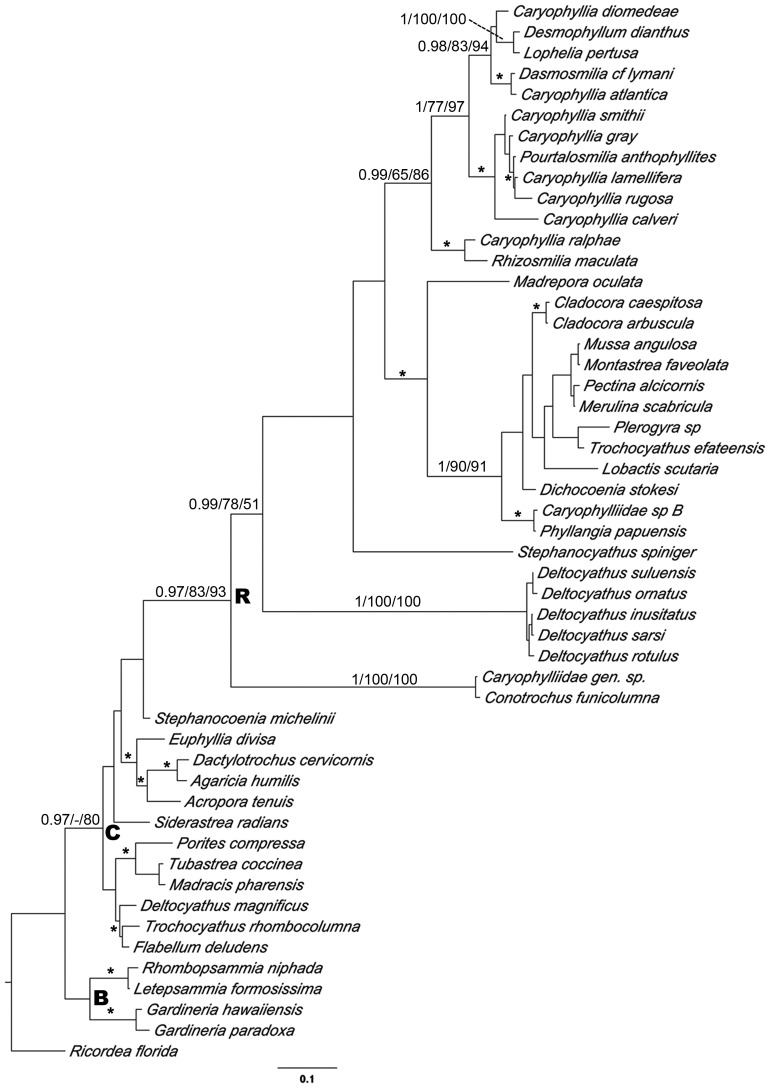
Relationship between *Desmophyllum dianthus* and principal taxa from Scleractinia families based on mitochondrial COI. Phylogenetic relationships among *D. dianthus* and representative species of the family Caryophylliidae. R, C and B indicate “robust”, “complex” and “basal” groups, respectively. The phylogenetic relationships were inferred by BI, MP and ML criteria (numbers show the Bayesian posterior probability and bootstrap supports given at branches, respectively). Stars indicate other well-supported clades (pp≥95; bootstrap >70).

The results of phylogenetic analyses under BI, ML and MP approaches are summarized in Fig. 1, Fig. 2 and Fig. S2. Phylogenetic analyses of each gene region were consistent and yielded the same tree topologies. Nuclear and mitochondrial DNA combined analyses provided the greatest resolution, after verifying their congruence through partition-homogeneity test (P = 0.26). The phylogenetic analyses indicated *D. dianthus* within the well-supported monophyletic “robust” group, consistent with previous analyses. No apparent genetic structure was found for *D. dianthus* sequences in any of the phylogenetic analyses, and the sequences consistently appeared as a polytomy (Fig. 1). Almost no inter-individual divergence was found among the sequences of this species, with a maximum of 0.93% divergence for the combined matrix. The closest related species to *D. dianthus* was *L. pertusa*, showing 2.25% and 4.80% maximum pairwise divergence with *D. dianthus* for mitochondrial DNA sequences (COI and 16S, respectively) and 0.26% and 2.10% for nuclear regions (28S and ITS, respectively); but no noteworthy divergences among most sequences of both species were found in 28S, ITS, and 16S data. In fact, the *D. dianthus*+*L. pertusa* clade was always highly supported by both bootstrap values and posterior probabilities. The phylogenetic relationships based on all utilized markers, as shown in Fig. 1, also fully supported *Caryophyllia calveri* as the sister group to *D. dianthus*+*L. pertusa*, with a high range of pairwise divergence values (1.98 and 9.25% for 28S and COI, respectively). *Caryophyllia smithii* and *Pourtalosmilia anthophyllites* completed the Caryophylliidae family cluster, but it could not be considered a clade as the position of *Cladocora caespitosa* was unresolved in the different phylogenetic analyses. *Montastrea faveolata*+*Mussa angulosa*, *Madracis mirabilis*, and *Madrepora oculata* completed the representative species of the scleractinian “robust” group. Depending on what gene was selected, the “complex” group appeared to be monophyletic or paraphyletic. Caryophylliidae species, which clustered in both groups, were not recovered as monophylies at the genus level or into putative clades as defined by previous studies [11], [50].

### Haplotype Network

**Figure 3:**
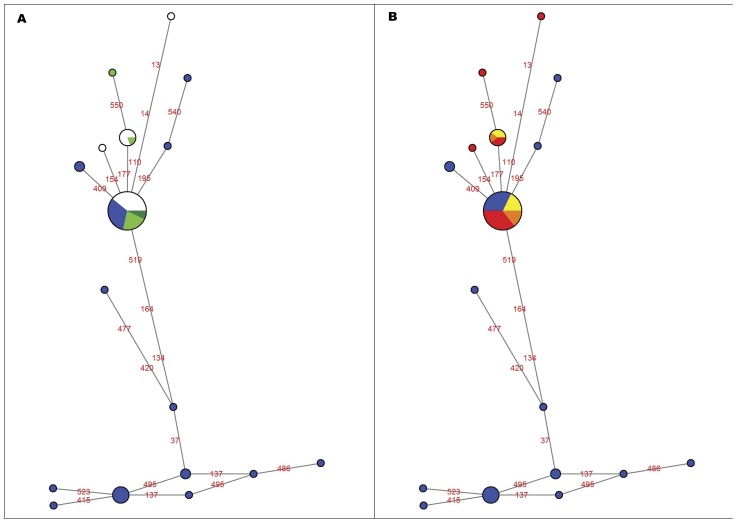
Haplotypes network. Parsimony network of internal transcribed spacer (ITS) ribosomal DNA sequence haplotypes of *Desmophyllum dianthus* belonging to Mediterranean Sea populations (from this study) and South Pacific Ocean populations (in blue; from Miller *et al.* [35], [36]). Sizes of the circles are proportional to the number of samples presenting such haplotype. Numbers indicate the variable positions. A) Network based on depth (white = shallow <600 m; light green = medium 600–1000 m; dark green = deep >1000 m). B) Network based on sampling area (red = Ionian Sea; orange = Adriatic Sea; yellow = Strait of Sicily).

Relationships within *D. dianthus* haplotypes were represented as a network (Fig. 3). Haplotype diversity (Hd) and nucleotide diversity (p) values were highest for COI sequences (0.86 and 0.0052, respectively); however, the ITS alignment was selected for the network analysis as more information existed in the literature for other populations.

The ITS network analysis (Fig. 3) was conducted considering gaps as missing positions. ITS sequences differed by a maximum of 12 substitutions. Sequences from Mediterranean and South Pacific specimens shared haplotypes and showed the same degree of difference as seen among sequences from Mediterranean samples (Fst = 0.37, p = 0.00). This apparent differentiation did not correspond to any clear geographic structure among populations distributed in regions separated by several thousands of kilometers. Geographic structure was also not found in 16S (Fst = 0.21, p = 0.00; Fig. S3), 28S or COI alignments (data not shown). Homologous nuclear and mitochondrial DNA sequences were utilized to perform neutrality tests to examine the presence of the evolutionary forces/processes occurring on Mediterranean corals from several locations. Negative Tajima's D and Fu's Fs values (Table 2) showed an excess of low frequency alleles and recent mutations, indicating that the Mediterranean population has been evolving randomly and is undergoing population size expansion and/or purifying selection.

**Table 2:** Measures of DNA polymorphism and neutrality tests (Tajima's D and Fu's Fs tests) for nuclear and mitochondrial DNA marker sequences of *Desmophyllum dianthus* specimens.

	Sequences	Sites	S	η	Haplotypes	Hd	p	D	p	Fs	p
*ITS*	83	643	11	11	7	0,14	0,0006	−2,27	0	−5,78	0,003
*28S*	79	758	9	9	8	0,17	0,0003	−2,26	0,01	−9,91	0
*16S*	35	279	16	17	4	0,17	0,0039	−2,45	0,01	0,74	0,245
*COI*	29	532	21	22	18	0,86	0,0052	−1,84	0,05	−11,21	0

S = segregating (polymorphic) sites; η = total number of substitutions; **Hd** = haplotype diversity; **p** = nucleotide diversity; D = Tajima's D value; Fs = Fu's Fs value; p = p value.

## Discussion

### Phylogenetic analyses

The three phylogenetic methodologies (ML, MP and BI), based on data from four molecular markers, all supported *D. dianthus* as clearly belonging to the “robust” clade, as previously implied by morphological analyses and molecular analyses of the nuclear 28S rDNA [12], [38], [50] and mitochondrial 16S rDNA and 12S rDNA sequences [10]. Surprisingly, in this study, no clear differentiation between *D. dianthus* and *L. pertusa* was found, in contrast to results from previous molecular and morphological supertree analyses [50]. Recently, Huang [53] grouped *D. dianthus*, *L. pertusa* and the genus *Caryophyllia* within an unresolved phylogenetic clade. In our molecular phylogeny, *L. pertusa* and *D. dianthus* not only grouped together in a resolved cluster, but also shared haplotypes for some DNA markers (e.g. ITS and COI). This fact illustrates a previously mentioned problem related to the selection of markers analyzed in corals thus far: mitochondrial DNA (i.e. 16S and COI) is less variable in Anthozoans compared to non-Anthozoans [52]–[56], and nuclear markers (i.e. ITS and 28S) often do not provide enough information for coral phylogenetic studies [57]–[61]. However, recent genome and transcriptome sequencing studies have shown that coral nuclear genomes have a high number of SNPs, which may represent promising future molecular markers [62], [63].

Furthermore, our results support the hypothesis that the morphological similarity of *D. dianthus* and *L. pertusa* reflects a close evolutionary relationship, and provide a new avenue of investigation to study the evolution of acquisition/loss of colonial/solitary life forms between closely related species (e.g. [9], [10], [12]).

The sister species of the *D. dianthus*+*L. pertusa* cluster in the combined analysis was *C. calveri*. These are the first molecular data reported for *C. calveri*, showing that it belongs to the “robust” clade (Fig. 1) and confirming its placement within *Caryophyllia* (Fig. 2 and Fig. S2). However, as also envisaged by Kitahara *et al.* [64], the supposed monophyly of this genus is disrupted by the inclusion of additional species from the same genus. Herein, our results confirm that *Caryophyllia* does not constitute a monophyletic group due to the presence of *Desmophyllum*, *Lophelia*, *Pourtalosmilia*, *Dasmosmilia*, *Solenosmilia, Crispatotrochus* and *Rhizosmilia* in the same clade. Numerous scleractinian molecular studies have highlighted the lack of agreement between taxonomic classification based on traditional morphological characters and evolutionary lineages recovered by molecular markers (e.g. [8], [11], [64], [65]), prompting a call for an in-depth taxonomic reassessment of the Scleractinia genera.

In this regard, the family Caryophylliidae represents a model case. Molecular analyses in which an adequate number of taxa were included support the splitting of this family into multiple different groups [49]. Le Goff Vitry *et al.* [39] sequenced mitochondrial 16S from 13 taxa identified as Caryophylliidae and found five distinct groups. Currently, one of these clades is now considered as belonging to the family Euphyllidae, but at least four clades of polyphyletic Caryophylliidae remain. Kerr [50] combined existing molecular and morphological phylogenies into a supertree summary, analyzed 61 taxa of caryophilliids species, and also recovered five clades of Caryophylliidae. Kitahara *et al.* [11] analyzed COI in 23 Caryophylliidae taxa and showed that the family is in nine lineages spread throughout the scleractinian phylogeny. This was also supported by a recent study by Huang [53].

In the present study, we analyzed data from four different molecular markers and also recovered the polyphyly of Caryophyllidae, consistent with previous studies. However, our results also showed a well-supported differentiation between the *D. dianthus*+*L. pertusa* clade and the *Caryophyllia* genus.

Two possible explanations related to the lack of phylogenetic signal from morphological diagnostic features in corals could account for our observations. One possibility is that the morphological characters used to date are homoplasious, while the other possibility is that the substitution rates of the genes used in this study cannot disentangle the evolutionary history of Scleractinia.

Current strategies for conducting large phylogenetic analyses focus on data combination using supertree and supermatrix methods. Despite their utility, there has been much debate about the relative merits of the best strategy when using sequence data from multiple genes or the utilization of evidence from different datasets, especially when some genes or characters have yet described for some species [66], [67 and references therein].

When such data are missing, both supertree and supermatrix strategies can lack statistical support and ignore uncertainties in the subtrees/matrices [66], [68], which sometimes can lead to a misinterpretation of the phylogenetic relationship among species. In fact, the lack of data due to the incomplete presence of genes for all of the species analyzed can yield irregular matrices. This could lead to inferring an erroneous phylogeny; for example, the surprise grouping of *D. dianthus*+*L. pertusa* into a clade, and the clear relationship between this clade and *C. calveri* and *C. smithii*, might have not been observed. Further studies that improve current methodologies or find alternative approaches are necessary to resolve “irresolvable” evolutionary questions.

### Haplotype Network

Previous studies [35], [36], [39], [69]–[74] have shown that the nuclear ITS region is more informative for distinguishing between geographically and bathymetrically isolated populations than either of the mitochondrial DNA regions. Patterns have been found within fjords and open slope regions for other deep-sea corals, such as for *L. pertusa* in the northeast Atlantic Ocean [75], suggesting that gene flow among geographically separated populations may be high. Costantini *et al.* [74] and Eytan *et al.* [73] reported depth as a potentially important physical factor and as an isolating mechanism for eurybathic species, which has also been demonstrated in other studies (e.g. [14], [15], [76], [77]). Other interesting results have been found by Miller *et al.* [35], where genetic differentiation among seamounts off Tasmania, Australia and the Auckland Islands was apparent only in the coral *D. dianthus* and not in other scleractinians or antipatharians. Nevertheless, in spite of the significant values found in Miller's *et al.* [35] study for certain genetic subdivision indices, the analyzed localities still share some common haplotypes. Miller *et al.* [36] provided evidence of statistically significant levels of genetic differentiation consistent with limited gene flow and isolation, and indicated that depth was a major component of such differentiation. The strongest pattern of depth stratification was found from ITS sequence data. The dynamics of the fluctuation of the oxygen-minimum zone (OMZ) and the shoaling of the aragonite saturation horizon (ASH) may act as barriers to gene flow in the deep sea, leading to speciation in marine invertebrates [78]–[81]. Such results can explain how geographically isolated populations from southeast Australia, New Zealand and Chile are genetically subdivided more by their stratigraphic bathymetry than by their geographic distances.

As previously mentioned, our samples were obtained from the Adriatic Sea (276–720 m), Ionian Sea (482–1102 m) and Strait of Sicily (403–850 m). The intermediate and deep water currents mainly characterizing the area are the Levantine Intermediate Water (LIW), Adriatic Deep Water (AdDW) and Aegean Deep Water currents (AeDW) (Fig. S1). The LIW circulates at approximately 200–600 m along the northeastern slope of the Ionian Sea, penetrating into the southern Adriatic Sea, and then continues along a slope as far as the Strait of Sicily, where most of it outflows into the Western Basin (at 400 m). The other two currents first accumulate in the troughs (1000–1500 m) over which they are formed (in the southern Adriatic and southern Aegean Seas, respectively) before outflowing through various openings (Fig. S1, Table S1) [69]. These water masses are depth-stratified and may represent two distinct bathymetric levels that could create depth structuring in species diversity and community composition.

Along more than 1000 km of the southeastern and southwestern Italian slopes in the Mediterranean Sea, most of the specimens analyzed shared common nuclear (i.e. ITS and 28S) and mitochondrial (i.e. 16S and COI) genotypes and/or haplotypes, suggesting high regional connectivity among deep-sea populations from the Adriatic Sea, Ionian Sea and Strait of Sicily.

The well recognized slow evolutionary rate of mitochondrial DNA in corals, estimated to be up to 10 times slower than relative nuclear DNA markers [53]–[56], may be responsible for the presence of limited numbers of mtDNA haplotypes across all populations. Surprisingly, nuclear data sequences provide no genetic differentiation among Mediterranean populations.

Physical connectivity of Mediterranean sites may be attributed to the principal currents in intermediate and deep waters [69], [70]. Moreover, planktonic *D. dianthus* larvae are thought to be retained within natal deep-water masses [36], and taken together with the patterns of LIW, AdDW, and AeDW currents flowing in these regions, we hypothesized that larvae should easily be able to disperse within the region along the southern Italian continental margin, thereby maintaining genetic connectivity among contiguous regions.

Instead, indices of genetic differentiation (F_ST_ = 0.37, p = 0.00) were found among sampling sites distributed in regions separated by supposedly clear geographic barriers, such as the Mediterranean Sea and South Pacific Ocean. Surprisingly, the network analyses also showed haplotypes being shared between these two areas. The occurrence of shared haplotypes between specimens from northern and southern hemispheres could indicate historical patterns of genetic diversity (current or recent gene flow, incomplete lineage sorting or retention of ancestral polymorphism), methodological bias (using genes or regions with a substitution rate inadequate to show divergence) or both (differences in the coalescence of these genes combined with populations divergence).

The extent of gene flow is correlated with reproductive traits, and understanding the processes that limit or promote dispersal in coral species can provide insights into how populations persist and evolve [82]. Although studies on deep-water coral reproduction are increasing, many life history aspects associated with coral reproductive strategies such as larval longevity, long-distance dispersal potential and mortality, among others, are still poorly understood. Even if it is not unusual for scleractinian species to have differing reproductive patterns [83], [84] we hypothesize that *D.dianthus* may have similar reproductive strategies as observed in other deep-water corals species [85]: broadcast spawners with lecithotrophic larvae. Since coral larvae are relatively poor swimmers, their dispersal distance largely depends on their longevity (i.e. maximum lifespan), settlement competence, larval survival and oceanographic factors (e.g. speed and direction of water currents) [86], [87]. Graham *et al.* [88] showed that larval longevities are much greater than previously reported (on the order of 200 days or more), and thus, should be sufficient to allow very long-distance dispersal. Moreover, this study [88] also provided strong support for high early and late mortality of coral larvae, suggesting that although the potential for rare long-distance dispersal events exists, most larvae do not survive long enough to be transported very far. Thus, the majority of successful recruitments are likely to involve settlement on natal or neighboring reefs, particularly given that most larvae become competent to settle quickly, within a few days after spawning [88]. In fact, a recently study on planktonic larval durations (PLDs) by Burgess *et al.* [89] suggested that extended PLDs could affect the dynamics of adult populations directly (via reductions in settlement density) and indirectly (via reductions in the post-settlement performance of individuals that experienced a metamorphic delay before settling). Considering the results of the above studies, current or recent gene flow is unlikely given the paleogeographic history of the areas involved in the present study. Some connections can be argued among the different South Pacific populations, even if the distances between them are considerable, but the Mediterranean Sea has been effectively a semi-closed sea since more than five million years ago [90]. Connections with southern Pacific populations are doubtful because of the great distance and possible oceanographic barriers between these biogeographic regions. As demonstrated by Nunes *et al.* [82], the combined effects of distance and physical oceanography are likely important isolating factors for coral populations. These barriers may be more permeable for other organisms whose ecologies and life histories permit dispersal at greater distances than those of corals [82]. Even after selecting the most variable DNA markers (ITS and COI), the hypothesis of continued gene flow among populations cannot be fully supported because differences of haplotype frequencies among populations exist. The shared haplotypes may be ancestral haplotypes that have been maintained over time in the two populations without continued gene flow [82]. Since reproduction patterns and larvae life strategies (e.g. high early mortality rates), and consequently population dynamics, can differ substantially among species [85], [88], together with the challenging (from an investigative point of view) habitat of CWCs, future studies using a wide variety of approaches (e.g. from molecular genetic to biophysical modeling) have to be developed to extensively study the evolutionary history of these deep-water organisms.

In light of the results obtained in this study, it is apparent that *D. dianthus* and *L. pertusa*, and by extension Caryophylliidae and Scleractinia, need further taxonomic revision due to the lack of taxonomic congruence with observed evolutionary relationships. Therefore, in-depth analyses of new morphological features and molecular markers are critically needed.

None of the nuclear and mitochondrial DNA regions utilized here were useful as exhaustive markers for population studies on *D. dianthus*, and therefore, we could not exclude the hypothesis that the three Mediterranean areas investigated in this study constitute a unique *D. dianthus* population. Furthermore, we could not confirm any genetic structure between populations in the northern and southern hemispheres.

If depth or water circulation are important factors driving isolation, finding adequate molecular markers and morphological characters that can truly show a lack of connectivity between populations at different depths and from different oceans with high statistical significance is an absolute priority. Markers with higher evolutionary rates may be more informative for resolving genetic relationships at different spatial scales and for providing information that reflects current gene flow patterns.

## Supporting Information

Figure S1 **Water masses and circulation in the central Mediterranean Sea, modified from Reithdorf, 2008.**
(XLS)

Figure S2 **Mitochondrial 16S rRNA phylogenetic tree.**
(XLS)

Figure S3 **Network of mitochondrial 16S rDNA sequences.**
(XLS)

Table S1 **Depth, method, and geographic coordinates of sampling locations.**
(XLS)

Table S2 **List of scleractinian taxa used with GenBank Accession Numbers.**
(XLS)
